# Otolaryngology Residency Match During the COVID-19 Pandemic

**DOI:** 10.7759/cureus.23258

**Published:** 2022-03-17

**Authors:** Afreen A Siddiqui, Alyssa Reese, Sarah Debs, Michele M Carr

**Affiliations:** 1 Otolaryngology, Jacobs School of Medicine and Biomedical Sciences, Buffalo, USA; 2 Otolaryngology, Virginia Commonwealth University, Richmond, USA

**Keywords:** residency match during pandemic 2020, residency match, nrmp, program director, otolaryngology residency, residency

## Abstract

Objective

To review changes made by otolaryngology residency program directors (PDs) during the 2020-2021 National Resident Matching Program (NRMP) match cycle and describe their attitudes toward the 2021-2022 match cycle.

Methods

Cross-sectional study using an anonymous 31-item online survey in Research Electronic Data Capture (REDCap) with questions regarding the 2020-2021 NRMP match. This survey was distributed to 125 PDs from Accreditation Council for Graduate Medical Education (ACGME)-accredited otolaryngology residency programs.

Results

Thirty-three PDs responded (26.4%). Of the PDs, 78.8% had an online info-session prior to the start of the cycle, and 30.3% reported that an increased number of applicants contacted them compared to the prior cycle. There were no changes made in Step 1 criteria (72.7%), and 81.8% reported no changes in interview selection. Of the PDs, 54.5% reported interviewing more candidates. Respondents reported a decreased cancellation rate (66.7%) and cost of recruiting (87.9%); 87.9% said that they did not change the way they developed their rank order list (ROL), and 84.8% reported matching at their usual level compared to prior years. Of the respondents, 42.4% reported making a change that was an overall improvement for their program. Of the PDs, 34.4% were unsure whether they would sustain virtual interviews in 2021-2022, 25% stated that they would not incorporate virtual interviews, and 40.7% stated that they would incorporate a virtual interview in some part of the cycle.

Conclusion

Otolaryngology PDs approached virtual interviewing in different ways. Despite the changes made, applicants can find comfort in knowing that match outcomes were perceived as typical by a majority of PDs.

## Introduction

Technological advances continue to change residency recruitment practices in the National Resident Matching Program (NRMP) residency match, and the coronavirus pandemic has significantly accelerated these changes. Traditionally, after the submission of applications, fourth-year medical students are offered in-person interviews that require them to travel to the prospective program site. In order to ensure minimal contact and transmission of the virus, as well as to stay within state and federal compliance, all residency interviews were conducted virtually during the 2020-2021 match cycle. Two new initiatives for otolaryngology applicants to express interest in specific residency programs, applicant signaling and Short Talks by Aspiring Residents in Otolaryngology (StarOto), were also started in 2020. Otolaryngology Preference Signaling allowed otolaryngology applicants to “signal” five programs they were most interested in attending, while the StarOto program allowed applicants to upload a video of themselves presenting an otolaryngology-related topic. Additionally, the year put social injustices faced by minorities to the forefront, as COVID-19 exposed major health inequities. With the match completed and spots filled, these changes pose many questions about the process, durability through subsequent years, and the perceived quality of match outcomes.

In comparison to other specialties, there are significantly fewer positions for prospective otolaryngology applicants. A total of 350 otolaryngology residency positions were available for both the 2020 and 2021 match, according to the NRMP data [1,2]. All 350 spots were filled in 2021, while only 348 were filled in 2020. This was not due to a lack of applicants, as there was an increase in otolaryngology residency applicants between the 2020 and 2021 cycles. A total of 559 applications were submitted for the 2021 cycle, compared to 505 during the 2020 cycle [1,2].

Historically, otolaryngology remains one of the most competitive fields in the NRMP, with successful match rates of 69.3% and 62.6% in 2020 and 2021, respectively [1,2]. Based on the rising trend in applicant numbers and the stagnant number of residency positions, it is clear that the competitiveness of the specialty is not changing. The purpose of our survey was to identify the changes that were made to the 2020-2021 match cycle and identify the residency programs’ perspectives on match outcomes. Ultimately, this study hopes to equip otolaryngology program directors (PDs) and future otolaryngology applicants with effective strategies as they continue to adapt to changes in the residency application cycle as a result of COVID-19.

## Materials and methods

PDs of Accreditation Council for Graduate Medical Education (ACGME)-accredited otolaryngology programs (n = 125) were asked via email to complete a 31-item survey regarding the 2020-2021 NRMP Residency Match. Study data were collected and managed using Research Electronic Data Capture (REDCap) electronic data capture tools hosted at the University at Buffalo. More information regarding REDCap can be accessed on the REDCap website [3].

The cross-sectional survey was distributed in May 2021 and was open for two months, with weekly email reminders sent to nonresponders. The survey consisted of five sections: social media outreach, interviewee selection, interview process, post-interview review and outcomes, and demographics. Data was recorded in REDCap and ultimately analyzed using Statistical Package for the Social Sciences (SPSS) version 27.0 (IBM Corp., Armonk, NY, USA). Frequencies were calculated, and means with 95% confidence intervals were determined when necessary. This study was approved by the University at Buffalo Institutional Review Board.

## Results

A total of 33 out of 125 otolaryngology PDs participated in the study, with a 26.4% response rate. The majority of the PDs were located in the Northeast (n = 10, 30.3%) and the South, including Puerto Rico (n = 10, 30.3%), and 36.4% (n = 12) had more than 10 years of experience (Table 1). PDs reported interviewing for 1-6 residency positions, with the majority interviewing for three positions (n = 9, 27.3%) (Table 1).

**Table 1:** Demographics

Demographics		Number of PDs (n (%))
Location of program		
	Northeast	10 (30.3)
	Midwest	6 (18.2)
	West	6 (18.2)
	South (including Puerto Rico)	10 (30.3)
	No response	1 (3)
Years as PD		
	<5 years	9 (27.3)
	6–10 years	11 (33.3)
	>10 years	12 (36.4)
	Unknown	1 (3)
Number of positions available for 2021 match cycle		
	1	2 (6.1)
	2	6 (18.2)
	3	9 (27.3)
	4	7 (21.2)
	5	4 (12)
	6	1 (3)
	Unknown	4 (12.1)

Changes in social media use and virtual outreach were reported. Of those who reported having a social media account, 14 (42.4%) reported creating a new social media account for the 2020-2021 interview cycle (Table 2), while six (18.2%) reported accounts that were created prior to the cycle. Virtual informational sessions prior to interview season were reported by 78.8% (n = 26) of PDs, and 21.2% (n = 7) reported production of an informational video (Table 2). The most frequently used social media accounts were Instagram (n = 13, 39.4%) and Twitter (n = 7, 21.2%) (Table 2).

**Table 2:** Changes in social media outreach

Social media use		Number of PDs (%)	
		Yes (n (%))	No (n (%))
Existence of a social media account prior to March 2020		13 (39.4)	20 (60.6)
Social media account created in 2020		14 (42.4)	6 (18.2)
Social media platforms use			
	Instagram	13 (39.4)	20 (60.6)
	Twitter	7 (21.2)	26 (78.8)
	Facebook	3 (9.1)	30 (90.9)
Changes in virtual interaction			
	Creation of applicant informational video	7 (21.2)	26 (78.8)
	Online info-session prior to interview season	26 (78.8)	7 (21.2)
	Virtual social event for selected interviewees	26 (78.8)	7 (21.2)

PDs also answered questions about changes that may have occurred in their process of selecting applicants for interview invitations. Step 1 criteria during the 2019-2020 cycle remained unchanged for the interview selection process for 72.7% (n = 24) of programs (Table 3). Twenty-seven PDs (81.8%) reported no changes in applicant interview selection procedures (Table 3). However, 51.5% (n = 17) reported changes in interviewee selection based on “signaling,” including two PDs reporting the use of signaling as a tiebreaker (Table 3). Only five (15.2%) reported viewing applicant StarOto videos. Of the PDs, 54.5% (n = 18) reported an increase in the number of interviewees, but the majority of PDs reported not changing the number of interviewers or the time interviewees spent with interviewers (Table 4). Compared to 2019-2020, 66.7% (n = 22) of PDs reported fewer interview cancellations (Table 4).

**Table 3:** Changes in interviewee selection *Change in applicant interview selection procedure: one responded more holistic review and five responded incorporated signaling as a tiebreaker (×2), signaling helped, only interviewed signals, and signals helped. **Change in Step 1 criteria: one responded not sure. ***More applicants reached out: nine responded not sure.

	Yes (n (%))	No (n (%))
Change in applicant interview selection procedure*	6 (18.2)	27 (81.8)
Viewed StarOto videos of applicants	5 (15.2)	28 (84.8)
Changed list of interviewees based on applicant signaling	17 (51.5)	16 (48.5)
Change in Step 1 criteria	8 (24.2)**	24 (72.7)
More applicants reached out by email this year compared to previous years	10 (30.3)	14 (42.4)***

**Table 4:** Interview logistics

	Yes, fewer/less (n (%))	Yes, more (n (%))	No, same (n (%))
Change in the number of interviewees	1 (3)	18 (54.5)	13 (39.4)
Change in the number of interviewers	5 (15.2)	4 (12.1)	24 (72.7)
Change in the time interviewees spent with interviewers - either the length or number of interviews	6 (18.2)	3 (9.1)	23 (69.7)
Interview cancellation rate compared to 2019–2020	22 (66.7)	0 (0)	10 (30.3)
Total cost of recruiting compared to 2019–2020	29 (87.9)	1 (3)	2 (6.1)

The cost of recruiting significantly decreased, according to the majority of participants (Table 4). Despite the virtual nature of the interview, items of value were still sent to interviewees by all but one PD. Items included meal vouchers (n = 7, 21.2%), imprinted items (n = 10, 30.3%), and academic items (n = 1, 3%). It was unclear if some programs sent out multiple items to applicants.

**Figure 1:**
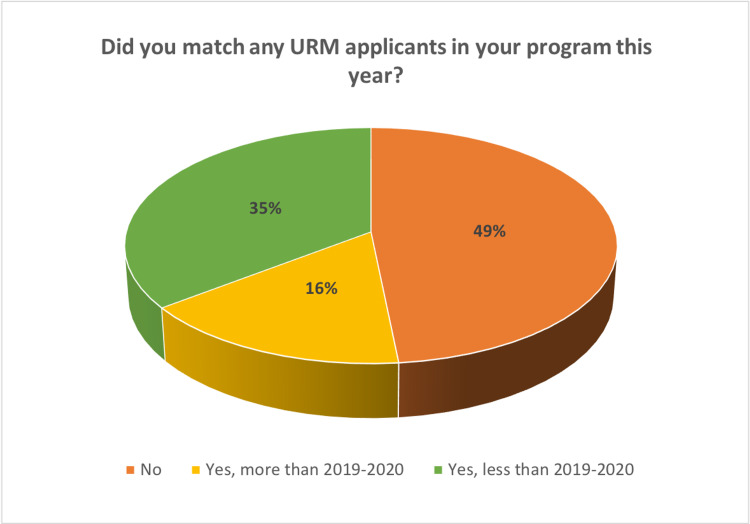
Matching outcomes for URM applicants

The process by which the PDs developed their rank order list (ROL) was unchanged in 87.9% (n = 29) of respondents, and 84.8% (n = 28) felt that they matched at their typical level on their ROL (Table 5). Of the respondents, 42.2% (n = 14) felt as though a change was made during the 2021-2020 interview cycle that was an overall improvement for their program (Table 6), and 48.4% (n = 16) of programs reported matching no candidate considered to be part of groups underrepresented in medicine, but 16.2% (n = 6) reported matching more such candidates than in 2019-2020 (Figure 1).

**Table 5:** Post-interview selection and match outcomes *ROL change: one respondent was unsure. **Participants were asked to specify what specific changes they employed that improved their program.

	Yes (n (%))	No (n (%))
Did you change the way you developed your ROL?	1 (3)	29 (87.9)*
Did your program match at your typical level on your ROL?	28 (84.8)	3 (9.1)
Did you make a change to this interview cycle that you think was an overall improvement for your program?	14 (42.4)**	18 (54.5)

**Table 6:** Changes made by PDs that proved beneficial

Beneficial changes made by PDs
Group virtual interviews
Real-time review and scoring system of interview candidates to utilize in the daily and final ranking
Increased virtual/online presence
Increased content on website
Virtual learning session prior to interviews
Targeting of underrepresented in medicine candidates for interview invites
Signaling
Shortened interview times
Held interviews on days other than Friday and Saturday
Virtual meet-and-greets
Pre-recorded information sessions

**Figure 2:**
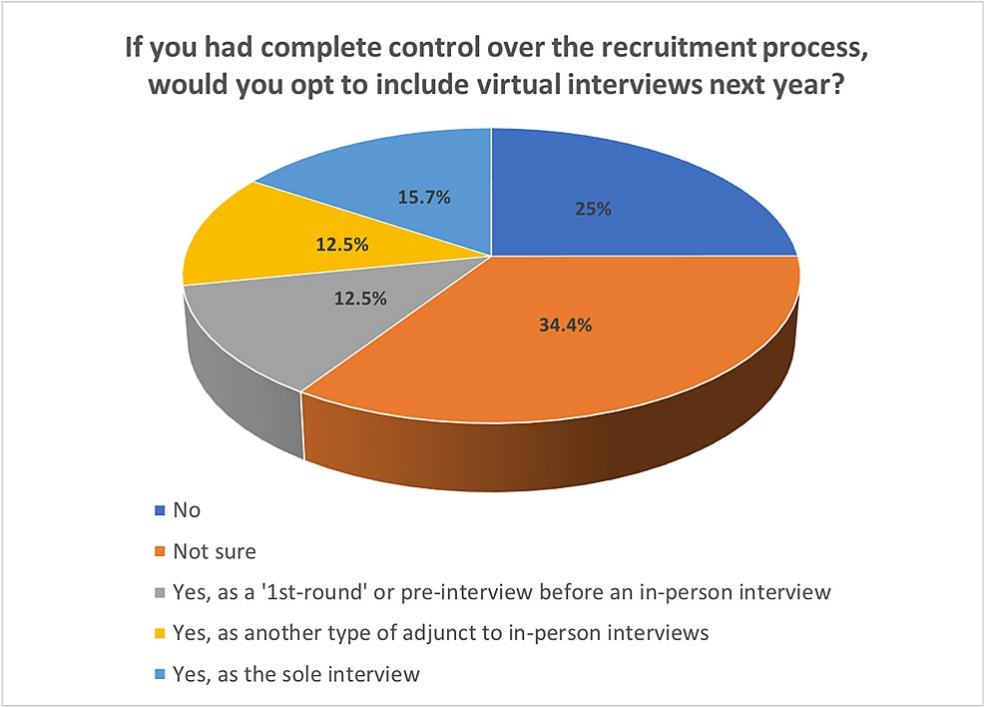
Role of virtual interviews in 2021-2022 match cycle

In terms of future changes in the application process, 40.7% (n = 13) of the respondents reported that if they had complete control over the recruitment process, then they would opt to include virtual interviews as either the sole interview, another type of interview adjunct to in-person ones, or a pre-interview before an in-person interview (Figure 2). Specific responses to this are shown in Table 6.

## Discussion

The applicant review process for residency is intricate and complex, with each program employing its own individual algorithm to select candidates for interviews and subsequent ranking. The unprecedented restrictions brought forth on travel and in-person meetings related to the COVID-19 pandemic resulted in modifications to this algorithm. Numerous sources speculated what this would mean for the match cycle; a pre-cycle survey of otolaryngology applicants showed that 54.1% felt less confident in matching [4]. We divided our questionnaire into the following categories: social media outreach, interviewee selection, interview process, post-interview review and outcomes, and demographics.

Otolaryngology residency programs have had an increasing social media presence over the last decade, with as many as 61% of programs having at least one social media profile [5]. Many of these social media accounts were established prior to the March 2020 pandemic in the United States. Goshtasbi et al. showed that a large majority of otolaryngology residency programs preferred Instagram and Twitter over Facebook and that 67.2% of those Instagram accounts and 20% of those Twitter accounts did not exist prior to March 2020 [6]. Our survey showed an even split between old and new social media accounts created after March 2020, with Instagram and Twitter making up a large percentage of utilized platforms. These social media accounts had a larger role for PDs, as well as applicants who no longer had the opportunity to physically assess compatibility to a program. A post-match applicant and PD survey conducted by the National Resident Matching Program (NRMP) showed that applicants were most concerned about determining if a program was a good fit based on web materials [7].

Otolaryngology Preference Signaling was a newly instituted concept that began in October 2020. The program allowed otolaryngology applicants to “signal” five programs they were most interested in attending, with the intention of facilitating matches based upon interest. This concept stems from the very successful signaling program employed within the field of business and finance, where job applicants similarly signal their interest to employers [8]. Updated match data from June 2021 shows that all programs participated in this initiative and that applicants who signaled were four times more likely to receive interviews from their signaled programs as compared to non-signaled programs [9]. We found that five of the six PDs who reported changes in applicant interview selection procedures cited signaling as the major change. With the rising number of applications for otolaryngology residency spots, signaling appears to be a valuable tool that will allow programs to prioritize those applicants who have a genuine interest in their programs and will allow applicants to separate themselves among a large pool. For PDs, this can serve as an additional tool in distinguishing applicants who have otherwise similar resumes. StarOto was another initiative that began its pilot program during the 2020-2021 match cycle; applicants were mentored by volunteer faculty members from institutions across the country as they created a video of themselves giving a short otolaryngology-related talk [10]. Our survey showed that only 15.2% of PDs viewed the StarOto videos of applicants.

Pre-interview communication is a more traditional avenue that many applicants take to express their interest in particular programs. Almost a third of PDs noticed an increase in applicants reaching out electronically to them compared to the prior cycle. Prospective applicants to otolaryngology programs surveyed last year expressed concern about a potential increase in the number of applications submitted per applicant, as well as the number of interviews attended per applicant [4]. This assumption, if true, would result in the cycle being much more competitive as fewer applicants would use more interview slots. This perception may explain why more than half of our respondents chose to interview more candidates than the previous year.

One major anticipated perk for the virtual cycle was the potential decrease in financial cost for applicants. According to the Association of American Medical Colleges (AAMC), the average amount an applicant spends on the entire interview season is about $4,000, ranging anywhere from $1,000 to $12,000 [11,12]. The post-match NRMP survey showed that over half of the applicants found the decreased cost of interviews to be a very important factor when it came to interview logistics. Other factors such as increased efficiency, the ability to attend more interviews, and the flexibility of interview dates were also deemed very important by a majority of respondents [7]. Cutting interview costs is a tremendous aid for those lacking the financial resources to attend distant in-person interviews, helping to create a more even playing field for a candidate pool with variations in socioeconomic status. Similar to interviewees, according to our data, the majority of PDs also reported a reduction in recruiting costs as a result of the virtual format of the interviews. Some estimates show average savings to be about $500 to $1,000 per interview per applicant [13].

This brings us to the main question - did the changes made during the 2020-2021 match cycle prevail? Only a quarter of PDs were against any form of virtual interviews in future cycles, indicating that virtual interviews may have a role in the future. This may be as adjuvant interviews, “pre-interviews,” or even the sole evaluation.

The need for virtual interviews allowed faculty to “think outside the box” and challenge their local traditions surrounding residency selection. One PD stated that the changes they made allowed their program to focus on recruiting underrepresented minority (URM) applicants. This may have reflected social and political events taking place in the USA in 2020 that brought racial and ethnic disparities to the forefront. As a result, many URM mentoring programs and travel grants were instituted in 2021 to encourage the recruitment of more URM otolaryngology applicants. As of 2020, out of all the surgical subspecialties, otolaryngology and thoracic surgery continue to have the lowest mean matriculation of URM residents [14].

With vaccination rates increasing and mandates becoming less strict, the future of the match remains uncertain. The Otolaryngology Program Directors Organization (OPDO) recommends that individual residency programs decide whether they would like to return to in-person interviews, continue with virtual interviews, or have a hybrid procedure during the 2021-2022 match cycle [15]. The ACGME, a member of the Coalition of Physician Accountability, has also recommended that all residency programs conduct their interviews virtually for the upcoming cycle [16]. At this time, it is unsure how many programs have opted out of virtual interviews, despite the recommendations. Nonetheless, it is evident that the virtual nature of the prior cycle presented many advantages to both applicants and PDs.

Limitations

This study only had a response rate of 26.4% of PDs. Thus, it may not be representative of the majority of otolaryngology programs. However, surveys addressing the same group have had similar response rates [17,18]. The margin of error is high, but despite that, there is still useful information for participants in the next otolaryngology match cycle. Additionally, the survey questionnaires were subjective, and respondents may have felt uncomfortable providing certain information about their programs, despite the anonymity of the survey.

## Conclusions

The virtual nature of the 2020-2021 otolaryngology match cycle presented some advantages to both applicants and PDs. Programs addressed the challenges in different ways. Economic advantages may have been important for particular programs and probably also for applicants with fewer financial resources. Despite the drastic changes that occurred during the cycle due to the pandemic, PDs felt that match outcomes were similar to prior years.
